# Blood and urinary cytokine balance and renal outcomes at orthopaedic surgery

**DOI:** 10.3389/fendo.2024.1441632

**Published:** 2024-12-26

**Authors:** William T. McBride, Mary Jo Kurth, Joanne Watt, Allister Irvine, Anna Domanska, Gavin McLean, John V. Lamont, Peter Fitzgerald, Mark W. Ruddock

**Affiliations:** ^1^ Department of Cardiac Anaesthesia, Belfast Health & Social Care Trust, Belfast, United Kingdom; ^2^ Clinical Studies Group, Randox Laboratories Ltd, Crumlin, United Kingdom

**Keywords:** acute kidney injury, biomarkers, cytokines, orthopaedic surgery, sTNFR1, sTNFR2, H-FABP, midkine

## Abstract

**Background:**

In patients undergoing orthopaedic trauma surgery, acute kidney injury (AKI) can develop post-operatively and is a major cause of increased mortality and hospital stay time. Development of AKI is associated with three main processes: inflammation, ischaemia-reperfusion injury (IRI) and hypoperfusion. In this study, we investigated whether ratios of urine and blood anti-inflammatory biomarkers and biomarkers of hypoperfusion, IRI and inflammation are elevated in patients who develop post-trauma orthopaedic surgery acute kidney injury (PTOS-AKI).

**Methods:**

Blood and urinary biomarkers of inflammation, hypoperfusion and IRI were analysed in 237 patients undergoing orthopaedic fracture surgery pre- and post-operatively. Biomarker ratios were compared between non-PTOS-AKI and PTOS-AKI patients.

**Results:**

Multiple inflammatory biomarkers were significantly elevated in PTOS-AKI patients compared to non-PTOS-AKI patients. When urine anti-inflammatory biomarkers were expressed as biomarker ratios with biomarkers of inflammation, hypoperfusion and IRI, multiple ratios were lower in PTOS-AKI patients. In contrast, blood anti-inflammatory biomarkers when expressed as ratios with blood proinflammatory biomarkers were elevated in PTOS-AKI patients.

**Discussion:**

Reductions in ratios of urine anti-inflammatory and proinflammatory biomarkers in PTOS-AKI patients suggest that the renal anti-inflammatory response is protective against the proinflammatory response in patients who do not develop PTOS-AKI. Detection of proinflammatory and anti-inflammatory biomarkers both pre- and post-operatively may be useful in detecting patients at risk of developing AKI after orthopaedic surgery.

## Introduction

Acute kidney injury (AKI) is a major complication of orthopaedic trauma surgery globally, particularly in the elderly (1). AKI is associated with heightened mortality (2), and increased length of hospital stay (3, 4) with healthcare resource implications, particularly for low-income countries where outcomes are poorer (5). Post-trauma orthopaedic surgery acute kidney injury (PTOS-AKI) risk factors include advanced age, pre-existing conditions e.g. chronic kidney disease and coronary artery disease (CAD) (2), male gender (6), hypoalbuminaemia and poor glycaemic control (7). Possible modifiable factors during the surgical procedure can also influence AKI risk including the choice of anaesthesia (spinal anaesthesia carries heightened risk of AKI) (1), use of peri-operative nephrotoxic drugs and excessive blood loss (3).

Spinal anaesthesia, pre-existing CAD or blood loss may contribute to AKI through increased possibility of peri-operative hypotension, a recognized risk factor of AKI in many surgical contexts, particularly if the mean arterial pressure (MAP) is < 65mmHg for longer than 5 minutes (8). If the hypotension has been significant, then a secondary process of ischaemia-reperfusion injury (IRI) -related AKI can develop (9). Blood loss also provides a further stimulus for AKI through activating the pro-coagulation compensatory process which drives the peri-operative proinflammatory response (10, 11), which has well recognized direct and indirect renotoxic effects (12).

There is merit in categorizing the many disparate clinical factors driving peri-operative AKI into their common renally injurious pathophysiological pathways, namely processes of hypoperfusion, IRI and proinflammation. Due to the complexity in quantifying the varying impacts of these processes for AKI in individual patients, attempts have been made to determine if biomarkers theoretically linked to processes of hypoperfusion (heart-type fatty acid-binding protein (H-FABP) and vascular endothelial growth factor (VEGF)), IRI (midkine), and proinflammation (proinflammatory cytokines) (13, 14) exhibit biopredictive significance in clinically detectable AKI. This approach has already shown some promise in the generation of a biomarker risk score in cardiac surgery-associated AKI (CS-AKI) (14) as well as orthopaedic fracture surgery (13).

Although the processes of proinflammation, hypoperfusion and IRI may be considered separately in the pathogenesis of AKI, it is important to note that hypoperfusion and IRI can lead to a secondary proinflammatory-mediated tubular injury which could exacerbate the direct tubular injury caused by hypoperfusion and IRI alone (15). This raises the question if there are intrarenal protective mechanisms which help to mitigate against direct and indirect tubulotoxic proinflammatory processes. In this context, attention has focused on an endogenous intrarenal anti-inflammatory response identified at cardiac surgery with (16) and without (17) cardiopulmonary bypass, as being potentially protective against proinflammatory-mediated peri-operative renal injury (18) as well as indirect proinflammatory injury due to hypoperfusion and IRI (14).

### Study objective and detailed hypotheses

Measurement of blood and urinary biomarker ratios were analyzed as surrogates for proinflammation, hypoperfusion and IRI (13) to examine the following three overarching hypotheses.

### Primary hypothesis

Previously we suggested that in cardiac surgery, an endogenous intrarenal anti-inflammatory response protects against the direct renal injurious effects of proinflammation as well as secondary proinflammatory renal injury indirectly arising from hypoperfusion and IRI (14). A similar mechanism may occur in patients undergoing orthopaedic surgery and therefore, our primary hypothesis is that the hypothesis 1.1–1.4 will be reduced in PTOS-AKI patients.


(1.1) $$\frac{urine\;anti-inflammatory\;biomarkers}{urine\;proinflammatory\;biomarkers}$$

(1.2) $$\frac{urine\;anti-inflammatory\;biomarkers}{blood\;proinflammatory\;biomarkers}$$

(1.3) $$\frac{urine\;anti-inflammatory\;biomarkers}{blood\;hypoperfusion\;biomarkers}$$

(1.4) $$\frac{urine\;anti-inflammatory\;biomarkers}{blood\;biomarkers\;of\;ischaemia\;reperfusion\;}$$

### Secondary hypothesis

Previously we hypothesized that the blood anti-inflammatory response could be used as a surrogate in patients undergoing cardiac surgery to reflect the underlying blood proinflammatory response, which is rapidly filtered from the blood and difficult to detect (14). In patients undergoing orthopaedic surgery, we investigated if a similar response occurred and, therefore, our secondary hypothesis is that the hypothesis 2 would also be reduced in PTOS-AKI:


(2) $$\frac{urine\;anti-inflammatory\;biomarkers}{blood\;anti-inflammatory\;biomarkers}$$

### Tertiary hypothesis

Finally, given that low molecular weight blood proinflammatory biomarkers rapidly leave the blood for the tubules and generate a large blood anti-inflammatory response which is sustained in cardiac surgery patients (14), we hypothesized that orthopaedic fracture surgery could also generate this response, as indicated by an increase in hypothesis 3 in patients with AKI:


(3) $$\frac{blood\;anti-inflammatory\;biomarkers}{blood\;proinflammatory\;biomarkers}$$

## Methods

### Study details

Details of the study were reported previously (13). Briefly, n=237 patients who were scheduled for open reduction and internal fixation of their fracture surgery were recruited to the study from the Fracture Unit of the Royal Victoria Hospital, Belfast, UK between May 2012 and August 2013. Written informed consent was obtained from all patients and the study was approved by the Office for Research Ethics Committee Northern Ireland, and the Royal Victoria Hospital Research Office Research Governance Committee. The study complied with Standards for Reporting Diagnostic Accuracy guidelines. The exclusion criteria for the study included: patients who were <18 years of age, those with a history of significant renal disease or those with pre-operative pre-trauma dialysis-dependent renal failure. Pre- and post-operative samples were available for 201/237 (84.8%) patients; patients without pre- and post-operative samples were excluded from the study.

Urine, serum and plasma samples were collected from all patients pre-operatively and post-operatively on day one. Patient samples were analyzed as described previously (13) by Randox Clinical Laboratory Services (RCLS, Antrim, UK, ISO17025 accredited).

### Definition of renal dysfunction

It was not possible to determine baseline estimated glomerular filtration rate (eGFR) prior to trauma in the patients. It was, therefore, assumed that the fracture patients had a history of normal renal function pre-operatively i.e., a baseline pre-injury eGFR of ≥60mls/min/1.73m^2^. Patients were defined as AKI positive if an eGFR value of <45mls/min/1.73m^2^ was recorded on any of the pre- and post-operative sampling days (days 0, 1, 2 and 5), in accordance with RIFLE classification of AKI where the risk stage is defined as a ≥25% drop in eGFR from a normal baseline (13, 19).

### Statistical analysis

All analyses were conducted using R (20). Significant biomarkers and biomarker ratios were determined using Mann–Whitney U test with a p value <0.05 considered significant. Area under receiver operator characteristic (AUROC) was used to determine the biomarker ratios with the best predictive ability at distinguishing between non-PTOS-AKI and PTOS-AKI patients. Least absolute shrinkage and selection operator (Lasso) method was used to select biomarker ratio combinations used in the models. For each biomarker and biomarker combination, AUROC, specificity, sensitivity, negative predictive value (NPV) and positive predictive value (PPV) were calculated.

## Results

Demographic data for the patients recruited to the study have been reported previously (13) and are also described in **Table 1**.

**Table 1:** Summary of patient clinical characteristics.

	Non PTOS-AKI (n=138) mean ± SD or number/total (%)	PTOS-AKI (n=63) mean ± SD or number/total (%)	p value
Patient demographics			
Age (years)	78.7 ± 10.9	85.5 ± 6.1	<0.001
Gender (female)	109/138 (79.0%)	42/63 (66.7%)	0.089
Comorbidities			
Dementia	15/138 (10.9%)	16/63 (25.4%)	0.015
Diabetes	11/138 (8.0%)	4/63 (6.3%)	0.907
Hypertension	38/138 (27.5%)	27/63 (42.9%)	0.046
Pre-surgery medications			
Hypertension medication	47/116 (40.5%)	29/51 (56.9%)	0.074
Operative method			
Intramedullary nailing	14/138 (10.1%)	1/63 (1.6%)	0.064
Hemiarthroplasty	60/138 (43.5%)	36/63 (57.1%)	0.100
Total hip replacement	10/138 (7.2%)	0/63 (0.0%)	0.065
Sliding hip screw	54/138 (39.1%)	26/63 (41.3%)	0.895
Intraoperative variables			
Packed red blood cells	6/115 (5.2%)	2/52 (3.8%)	1.000
Platelet bags	4/115 (3.5%)	2/52 (3.8%)	1.000
Fresh frozen plasma	0/115 (0.0%)	1/52 (1.9%)	0.683
Phenylephrine	19/115 (16.5%)	14/52 (26.9%)	0.176
Post operative variables			
Fresh frozen plasma	0/115 (0.0%)	1/52 (1.9%)	0.683
Packed red blood cells	34/115 (29.6%)	16/52 (30.8%)	1.000
Other variables			
Time between presentation and surgery (days)	2.1 ± 1.5	2.5 ± 2.0	0.138
Hospital stay (days)	9.8 ± 7.9	12.0 ± 8.3	0.020
Operation time (minutes)	53.8 ± 19.1	52.4 ± 18.4	0.636

PTOS-AKI, post-trauma orthopaedic surgery acute kidney injury. For additional information see previous publication (13).

### Biomarker changes in PTOS-AKI patients

The current study demonstrated that there were significant changes pre- and post-operatively in biomarker levels in patients who developed PTOS-AKI (**Table 2**).

**Table 2:** Biomarker levels in non-PTOS-AKI and PTOS-AKI patients.

Pre- or post-op	Biomarker Type	Biomarker	Non-PTOS-AKI (mean ± SD)	PTOS-AKI (mean ± SD)	p-value	Change
Pre-operative	Blood anti-inflammatory	**sTNFR1 (Serum)**	0.719 ± 0.312 (n=119)	0.972 ± 0.352 (n=55)	**<0.001**	Up
		**sTNFR2 (Serum)**	0.895 ± 0.777 (n=119)	1.185 ± 0.810 (n=55)	**0.004**	Up
	Blood hypoperfusion	**H-FABP (Serum)**	12.700 ± 13.012 (n=125)	18.872 ± 19.436 (n=58)	**<0.001**	Up
		VEGF (Plasma)	55.152 ± 53.677 (n=125)	53.469 ± 35.581 (n=59)	0.680	Down
	Blood proinflammatory	**NGAL (Plasma)**	802.965 ± 421.030 (n=125)	1136.045 ± 400.037 (n=59)	**<0.001**	Up
		**IL-12p40 (Serum)**	399.778 ± 249.068 (n=120)	517.844 ± 274.023 (n=55)	**0.004**	Up
		**MIP-1α (Plasma)**	5.473 ± 3.161 (n=125)	8.032 ± 9.990 (n=59)	**0.009**	Up
		**TNFα (Plasma)**	3.925 ± 7.085 (n=125)	4.195 ± 3.108 (n=59)	**0.019**	Up
		**MCP-1 (Plasma)**	187.968 ± 89.065 (n=125)	239.820 ± 172.391 (n=59)	**0.035**	Up
		IL-6 (Plasma)	77.165 ± 134.952 (n=125)	75.001 ± 87.130 (n=59)	0.054	Down
		IP-10 (Serum)	138.983 ± 125.976 (n=120)	149.159 ± 96.162 (n=55)	0.113	Up
		IL-8 (Plasma)	13.735 ± 42.301 (n=125)	10.754 ± 20.489 (n=59)	0.179	Down
		sIL2Rα (Serum)	0.261 ± 0.415 (n=119)	0.259 ± 0.213 (n=55)	0.379	Down
	Blood IRI	**Midkine (Serum)**	2093.636 ± 2199.370 (n=86)	2618.793 ± 2008.198 (n=42)	**0.035**	Up
	Urine anti-inflammatory	**sTNFR1 (Urine)**	3.034 ± 3.350 (n=119)	3.391 ± 2.215 (n=52)	**0.004**	Up
		sTNFR2 (Urine)	4.484 ± 4.090 (n=119)	4.802 ± 2.962 (n=51)	0.064	Up
		IL-1Ra (Urine)	5087.634 ± 7327.835 (n=110)	3699.390 ± 4793.242 (n=52)	0.610	Down
	Urine proinflammatory	IL-12p40 (Urine)	5.815 ± 8.400 (n=119)	7.315 ± 13.776 (n=52)	0.167	Up
		IP-10 (Urine)	61.395 ± 158.784 (n=119)	24.519 ± 44.659 (n=51)	0.191	Down
		NGAL (Urine)	318.949 ± 341.539 (n=110)	335.157 ± 371.330 (n=52)	0.810	Up
Post-operative	Blood anti-inflammatory	**sTNFR1 (Serum)**	0.942 ± 0.391 (n=101)	1.373 ± 0.444 (n=46)	**<0.001**	Up
		**sTNFR2 (Serum)**	1.127 ± 0.785 (n=101)	1.774 ± 0.878 (n=46)	**<0.001**	Up
	Blood hypoperfusion	**H-FABP (Serum)**	23.558 ± 16.980 (n=108)	51.897 ± 30.185 (n=48)	**<0.001**	Up
		VEGF (Plasma)	59.830 ± 56.264 (n=106)	53.117 ± 25.637 (n=52)	0.810	Down
	Blood proinflammatory	**NGAL (Plasma)**	786.655 ± 389.232 (n=106)	1241.043 ± 386.548 (n=52)	**<0.001**	Up
		**IL-12p40 (Serum)**	379.728 ± 241.892 (n=102)	459.888 ± 237.941 (n=46)	**0.021**	Up
		**MIP-1α (Plasma)**	212.962 ± 109.229 (n=106)	268.180 ± 161.446 (n=52)	**0.003**	Up
		**TNFα (Plasma)**	5.602 ± 14.418 (n=106)	4.835 ± 3.676 (n=52)	**0.002**	Down
		**MCP-1 (Plasma)**	6.036 ± 3.523 (n=106)	7.765 ± 4.068 (n=52)	**0.003**	Up
		**IL-6 (Plasma)**	1889.705 ± 1997.346 (n=86)	2639.552 ± 1777.919 (n=42)	**0.001**	Up
		IP-10 (Serum)	154.507 ± 156.851 (n=102)	156.275 ± 148.866 (n=46)	0.530	Up
		IL-8 (Plasma)	16.366 ± 56.914 (n=106)	12.097 ± 8.975 (n=52)	0.068	Down
		**sIL2Rα (Serum)**	0.260 ± 0.359 (n=101)	0.300 ± 0.233 (n=46)	**0.040**	Up
	Blood IRI	**Midkine (Serum)**	175.485 ± 165.901 (n=106)	277.953 ± 228.448 (n=52)	**0.001**	Up
	Urine anti-inflammatory	**sTNFR1 (Urine)**	5.282 ± 4.198 (n=106)	6.811 ± 3.871 (n=52)	**0.001**	Up
		sTNFR2 (Urine)	6.173 ± 4.138 (n=106)	7.179 ± 3.704 (n=52)	0.068	Up
		IL-1Ra (Urine)	7447.695 ± 8083.352 (n=101)	8398.598 ± 9165.445 (n=52)	0.449	Up
	Urine proinflammatory	IL-12p40 (Urine)	8.726 ± 24.412 (n=106)	8.678 ± 14.547 (n=52)	0.389	Down
		**NGAL (Urine)**	453.076 ± 287.406 (n=101)	606.845 ± 346.838 (n=52)	**0.008**	Up
		IP-10 (Urine)	128.587 ± 219.134 (n=106)	72.649 ± 101.098 (n=52)	0.528	Down

Significant biomarkers and p- values are bolded. Direction of biomarker level change in PTOS-AKI patients illustrated by ‘Up’ or ‘Down’. H-FABP, Heart-type fatty acid-binding protein; IL-1Ra,Interleukin-1 receptor antagonist; IL-6, Interleukin-6; IL-8, Interleukin-8; IL-12p40, Interleukin-12 subunit p40; IP-10, Interferon gamma-induced protein-10; IRI, ischaemia-reperfusion injury; MCP-1, Monocyte chemotactic protein-1; MIP-1a, Macrophage inflammatory protein-1a; n, number of patients; NGAL, Neutrophil gelatinase-associated lipocalin; Post-op, Postoperative; Pre-op, Preoperative; PTOS-AKI, post trauma orthopaedic surgery acute kidney injury; SD, standard deviation; sIL2Ra, Soluble interleukin-2 receptor alpha; sTNFR1, Soluble tumor necrosis factor receptor 1; sTNFR2, Soluble tumor necrosis factor receptor 2; TNFα, Tumor necrosis factor alpha; VEGF, Vascular endothelial growth factor.

Pre-operatively, there were 10/20 (50%) biomarkers which were significantly elevated in patients with PTOS-AKI (**Table 2**): sTNFR1 (serum and urine), sTNFR2 (serum), H-FABP (serum), NGAL (plasma), IL-12p40 (serum), MIP-1α (plasma), TNFα (plasma), MCP-1 (plasma), and midkine (serum). Post-operatively, there were 12/20 (60%) biomarkers which were significantly elevated in patients with PTOS-AKI (**Table 2**): sTNFR1 (serum and urine), sTNFR2 (serum), H-FABP (serum), NGAL (plasma and urine), IL-12p40 (serum), MIP-1α (plasma), MCP-1 (plasma), midkine (serum), IL-6 (plasma), and sIL-2Rα (serum). In contrast, only TNFα (serum) was significantly lower in patients who developed PTOS-AKI.

### Ratio differences in PTOS-AKI patients

**Figure 1:**
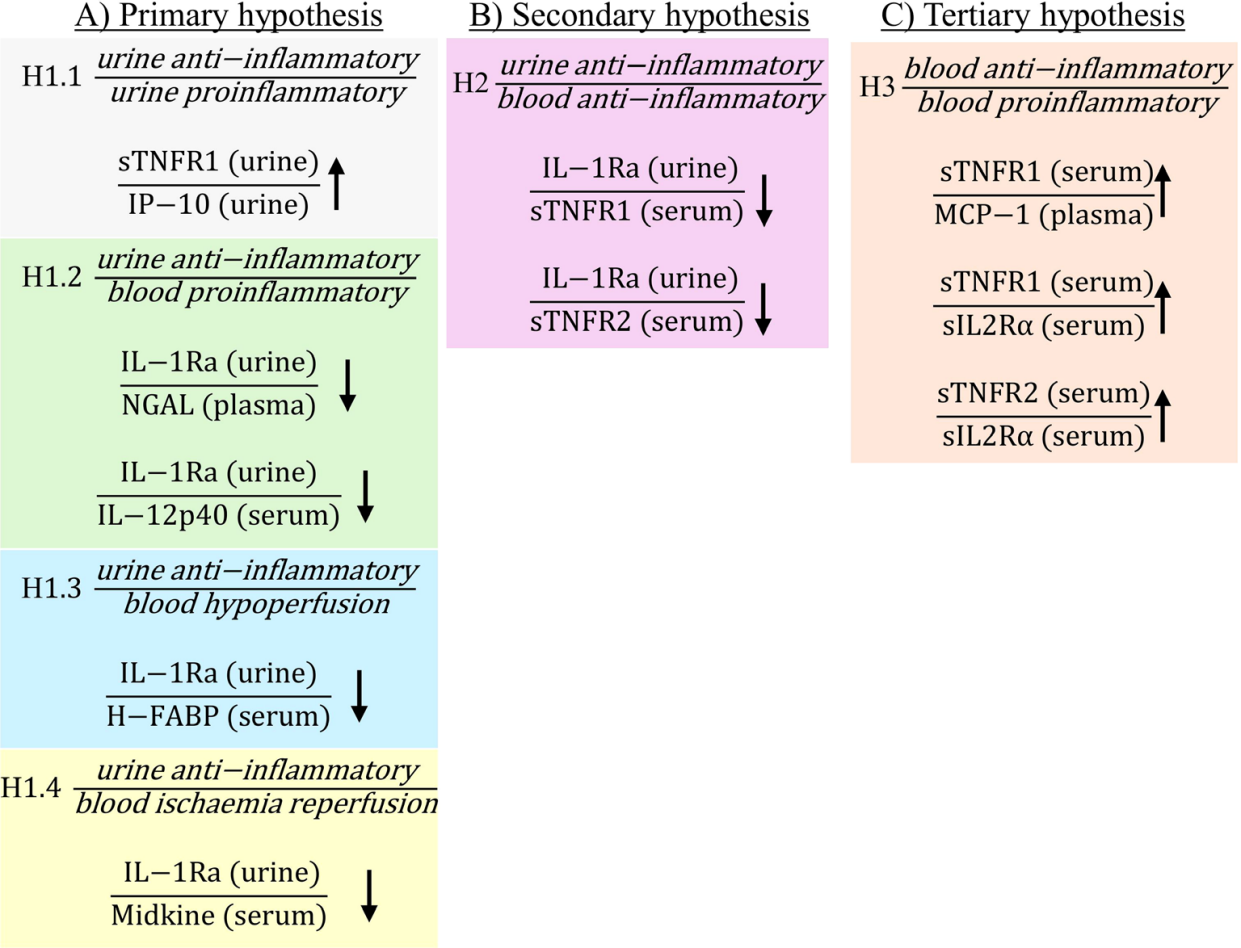
Statistically significant ratios of biomarkers pre-operatively between non-PTOS-AKI and PTOS-AKI patients according to the primary **(A)**, secondary **(B)** and tertiary **(C)** hypotheses. Full list of biomarker ratios including non-significant ratios are shown in **Supplementary Table S1**. Arrows indicate direction of change in biomarker ratios in PTOS-AKI patients.

**Figure 2:**
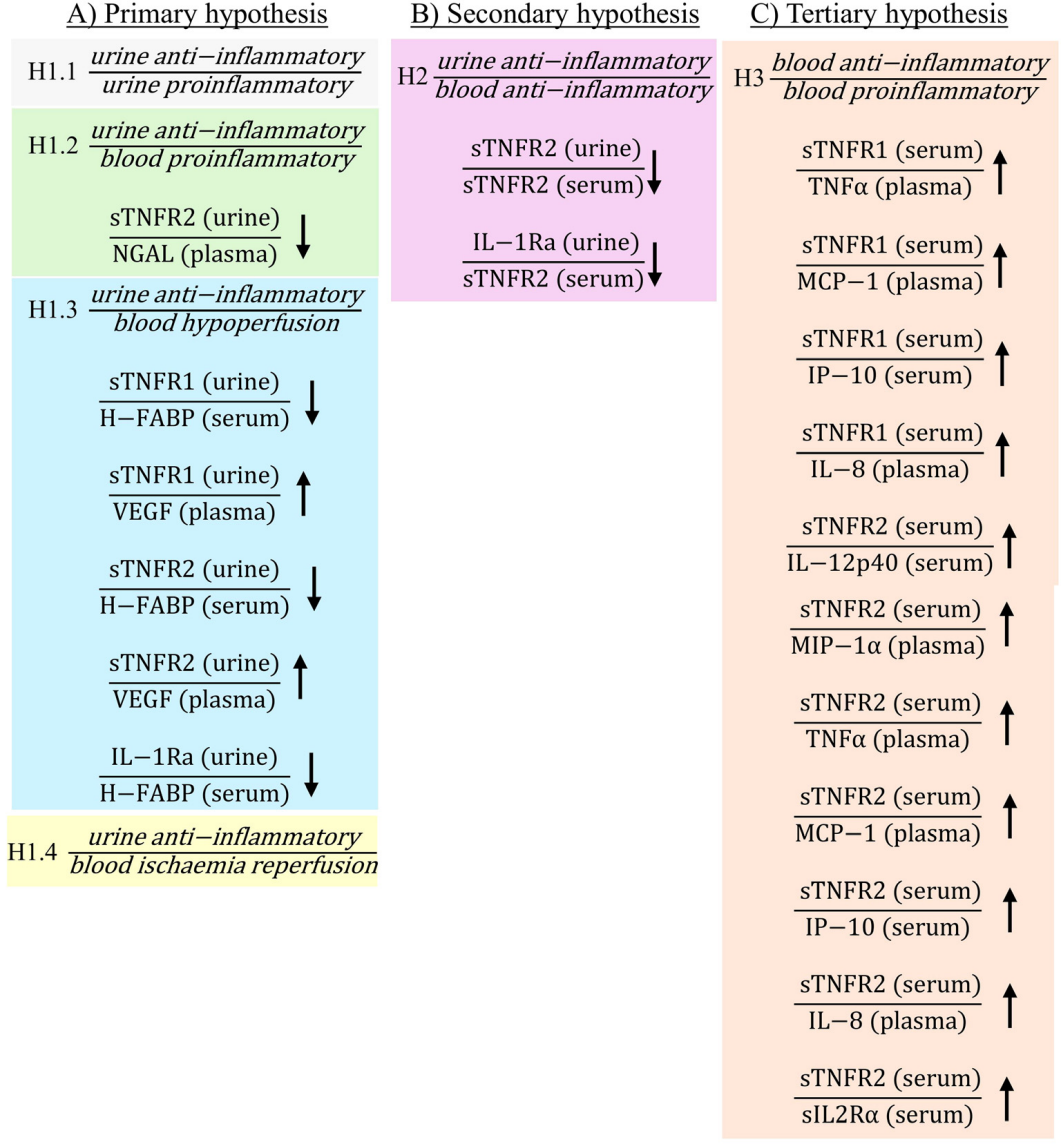
Statistically significant ratios of biomarkers post-operatively between non-PTOS-AKI and PTOS-AKI patients according to the primary **(A)**, secondary **(B)** and tertiary **(C)** hypotheses. Full list of biomarker ratios including non-significant ratios are shown in **Supplementary Table S1**. Arrows indicate direction of change in biomarker ratios in PTOS-AKI patients.

The current study demonstrated that there were significant changes in biomarker ratios both pre- and post-operatively in patients who developed PTOS-AKI (**Figures 1**, **2**; non-significant ratios shown in **Supplementary Table S1**). Pre-operatively there were 10 ratios which were significantly different in PTOS-AKI patients (**Figure 1**). Post-operatively there were 19 ratios which were significantly different in PTOS-AKI patients (**Figure 2**).

There were multiple ratios corresponding to the primary hypothesis which were significantly different in PTOS-AKI patients. When comparing urinary anti-inflammatory biomarkers to urinary proinflammatory biomarkers for hypothesis H1.1, only one ratio involving sTNFR1 was significantly higher in PTOS-AKI patients pre-operatively (**Figure 1A**). There were no significantly different ratios between patients post-operatively (**Figure 2A**). For hypothesis H1.2, pre-operatively, two ratios involving IL-1Ra were significantly lower in PTOS-AKI patients (**Figure 1A**). Post-operatively, only one ratio involving sTNFR2 was significantly lower in PTOS-AKI patients (**Figure 2A**). When comparing urinary anti-inflammatory biomarkers to blood biomarkers of hypoperfusion (H-FABP and VEGF), for hypothesis H1.3, there were six biomarker ratios which were significantly different in PTOS-AKI patients. Pre-operatively, one ratio was significantly lower in PTOS-AKI patients (**Figure 1A**). Post-operatively, three ratios were significantly lower in PTOS-AKI patients while two ratios were significantly higher in PTOS-AKI patients (**Figure 2A**). Finally, when comparing urinary anti-inflammatory biomarkers to a blood biomarker of IRI (midkine), for hypothesis H1.4, only one ratio was significantly lower pre-operatively in PTOS-AKI patients (**Figure 1A**). There were no significant differences between any of the ratios post-operatively (**Figure 2A**).

In the secondary hypothesis (H2), multiple ratios were significantly different. When comparing urinary anti-inflammatory to blood anti-inflammatory biomarkers, four ratios of biomarkers were significantly different in PTOS-AKI patients. Pre-operatively, two ratios involving IL-1Ra were significantly lower in PTOS-AKI patients (**Figure 1B**). Post-operatively, two ratios involving sTNFR2 were significantly lower in PTOS-AKI patients (**Figure 2B**).

For the tertiary hypothesis (H3), when comparing blood anti-inflammatory to blood proinflammatory biomarkers, 14 ratios of biomarkers were significantly different in PTOS-AKI patients. Pre-operatively, three biomarker ratios were significantly elevated in PTOS-AKI patients (**Figure 1C**). Post-operatively, eleven biomarker ratios were significantly elevated in PTOS-AKI patients (**Figure 2C**).

### Logistic regression

**Figure 3:**
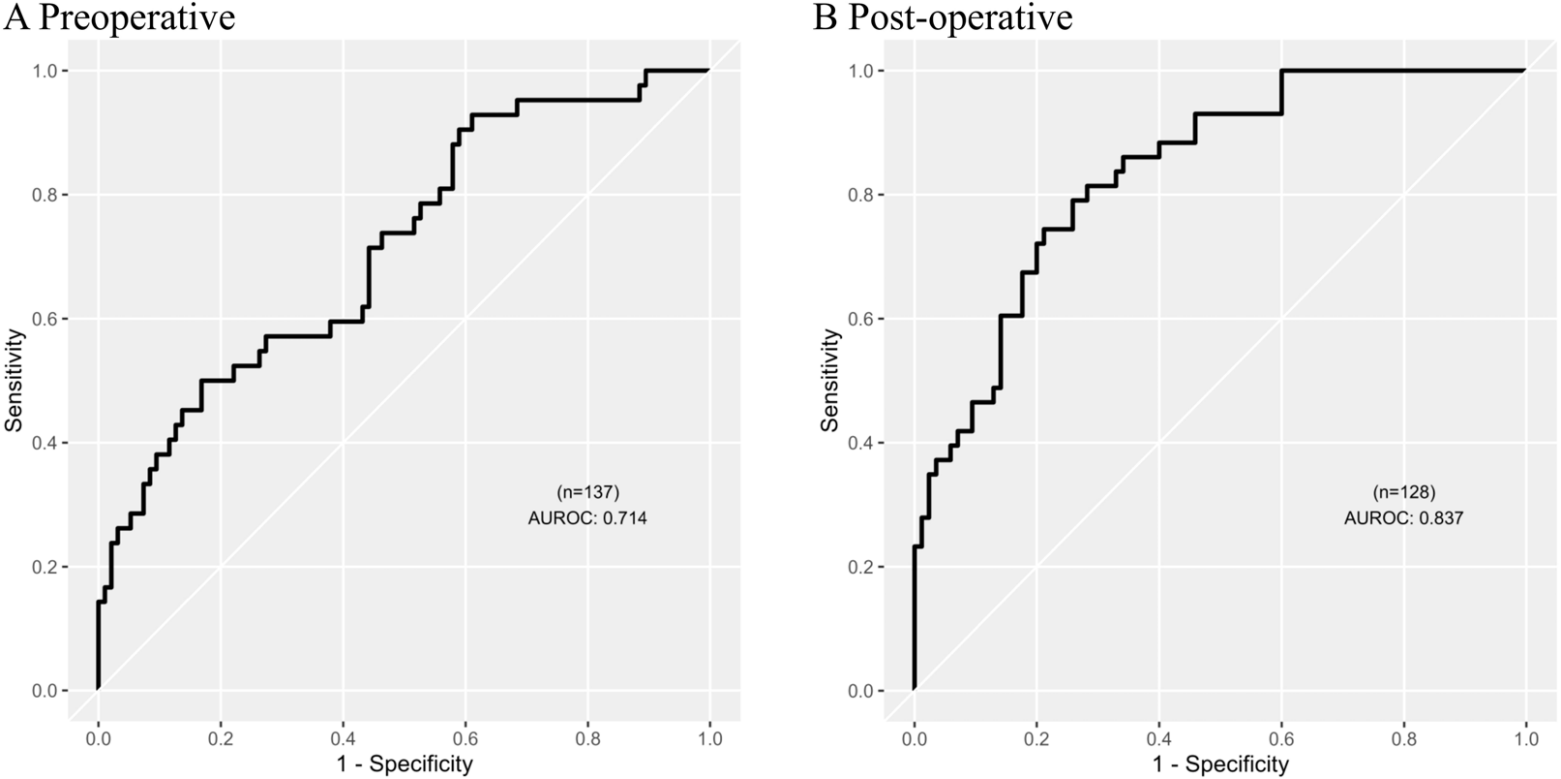
Logistic regression models determined by least absolute shrinkage and selection operator (Lasso) method with the highest predictive ability to stratify patients at risk of post-trauma orthopaedic surgery acute kidney injury (PTOS-AKI). **(A)** Pre-operative probability model of five biomarker ratios (**Table 3**) obtained by Lasso regression (AUROC 0.714). **(B)** Post-operative probability model of five biomarker ratios (**Table 3**) obtained by Lasso regression (AUROC 0.837). Full list of biomarker ratios modelled are shown in **Supplementary Table S2**.

Pre-operatively, a ratio of urinary anti-inflammatory IL-1Ra and serum hypoperfusion H-FABP gave the highest individual ratio AUROC of 0.642 (**Table 3**). Post-operatively, the ratio of urinary anti-inflammatory sTNFR2 and serum hypoperfusion H-FABP gave the highest individual ratio AUROC of 0.705 (**Table 3**). Lasso regression identified various combinations of ratios which could pre- and post-operatively stratify individuals at risk of developing PTOS-AKI; pre-operatively, a model of five ratios (**Table 3**) gave an AUROC of 0.714 (**Figure 3A**); post-operatively, a model of five ratios (**Table 3**) gave an AUROC of 0.837 (**Figure 3B**).

**Table 3:** Highest predictive biomarker ratios to identify patients at risk of developing PTOS-AKI.

	No. of ratios	Biomarker ratio (s)	AUROC (CI)	Sensitivity	Specificity
Pre-operative	1	$\frac{\text{IL}-1\text{Ra }\left(\text{urine}\right)}{\text{H}-\text{FABP }\left(\text{serum}\right)}$	0.642 (0.549-0.734)	56.3%	70.0%
	2	$\frac{\text{sTNFR1 (serum)}}{\text{sIL2Rα (serum) }}$ + $\frac{\text{IL-1Ra (urine)}}{\text{NGAL (plasma) }}$	0.695 (0.603-0.788)	60.0%	71.8%
	5	$\frac{\text{sTNFR}1\;\left(\text{serum}\right)}{\text{TNFα }\left(\text{plasma}\right)}$ + $\frac{\text{sTNFR}1\;\left(\text{serum}\right)}{\text{sIL}2\text{Rα }\left(\text{serum}\right)}$ + $\frac{\text{sTNFR}1\;\left(\text{urine}\right)}{\text{IP}-10\;\left(\text{urine}\right)}$ + $\frac{\text{IL}-1\text{Ra }\left(\text{urine}\right)}{\text{NGAL }\left(\text{plasma}\right)}$ + $\frac{\text{IL}-1\text{Ra }\left(\text{urine}\right)}{\text{H}-\text{FABP }\left(\text{serum}\right)\;}$	0.714 (0.621-0.808)	57.1%	72.6%
Post-operative	1	$\frac{\text{sTNFR}2\;\left(\text{urine}\right)}{\text{H}-\text{FABP }\left(\text{serum}\right)\;}$	0.705 (0.620- 0.790)	65.2%	71.1%
	2	$\frac{\text{sTNFR}2\;\left(\text{urine}\right)}{\text{H}-\text{FABP }(\text{serum})\;}$ + $\frac{\text{sTNFR}2\;\left(\text{serum}\right)}{\text{sIL}2\text{Rα }\left(\text{serum}\right)\;}$	0.764 (0.685- 0.842)	80.0%	65.2%
	3	$\frac{\text{IL}-1\text{Ra }\left(\text{urine}\right)}{\text{VEGF }\left(\text{plasma}\right)\;}$ + $\frac{\text{sTNFR}2\;\left(\text{urine}\right)}{\text{H}-\text{FABP }\left(\text{serum}\right)\;}$ + $\frac{\text{sTNFR}2\;\left(\text{serum}\right)}{\text{sIL}2\text{Rα }\left(\text{serum}\right)}$	0.813 (0.740- 0.886)	76.7%	71.8%
	4	$\frac{\text{IL}-1\text{Ra }\left(\text{urine}\right)}{\text{VEGF }\left(\text{plasma}\right)\;}$ + $\frac{\text{sTNFR}2\;\left(\text{serum}\right)}{\text{sIL}2\text{Rα }\left(\text{serum}\right)\;}$ + $\frac{\text{sTNFR}2\;\left(\text{urine}\right)}{\text{H}-\text{FABP }\left(\text{serum}\right)\;}$ + $\frac{\text{sTNFR}2\;\left(\text{serum}\right)}{\text{IP}-10\;\left(\text{serum}\right)}$	0.829 (0.758- 0.900)	74.4%	78.8%
	5	$\frac{\text{IL}-1\text{Ra }\left(\text{urine}\right)}{\text{VEGF }\left(\text{plasma}\right)\;}$ + $\frac{\text{sTNFR}2\;\left(\text{urine}\right)}{\text{H}-\text{FABP }\left(\text{serum}\right)\;}$ + $\frac{\text{sTNFR}2\;\left(\text{serum}\right)}{\text{sIL}2\text{Rα }\left(\text{serum}\right)\;}$ + $\frac{\text{sTNFR}2\;\left(\text{serum}\right)}{\text{MCP}-1\;\left(\text{plasma}\right)\;}$ + $\frac{\text{sTNFR}2\;\left(\text{serum}\right)}{\text{IP}-10\;\left(\text{serum}\right)\;}$	0.837 (0.768- 0.906)	74.4%	78.8%

Full list of biomarker ratios modelled are shown in **Supplementary Table S2**. AUROC, area under receiver operating characteristic; CI, confidence interval; H-FABP, Heart-type fatty acid-binding protein; IL-1Ra, Interleukin-1 receptor antagonist; IP-10, Interferon gamma-induced protein-10; MCP-1, Monocyte chemotactic protein-1; NGAL, Neutrophil gelatinase-associated lipocalin; PTOS-AKI, post trauma orthopaedic surgery acute kidney injury; sIL2Ra, Soluble interleukin-2 receptor alpha; sTNFR1, Soluble tumor necrosis factor receptor 1; sTNFR2, Soluble tumor necrosis factor receptor 2; TNFα, Tumor necrosis factor alpha; VEGF, Vascular endothelial growth factor.

## Discussion

Our primary hypothesis that an anti-inflammatory response protects against the renal injurious effects of proinflammation was partially confirmed by the demonstration that in patients with PTOS-AKI there were significant reductions in some ratios pre- and post-operatively in hypotheses H1.2, H1.3 and H1.4. Intrarenal proinflammatory-mediated injury can arise from direct renotoxic effects of proinflammatory biomarkers. Suggested mechanisms for this include direct proapoptotic effects as described for TNFα (21) as well as secondary tubular cell injury through inducible nitric oxide (NO) synthase induction which causes NO-mediated injury (22). However, the rapid intratubular removal of some filtered proinflammatory biomarkers such as TNFα and IL-1β as suggested by Bocci et al. (23) makes it impracticable to measure these filtered proinflammatory biomarkers in urine. For this reason, estimating the urinary proinflammatory response relies on the indirect methods of measuring plasma proinflammatory biomarkers prior to their filtration into the glomerulus. In this study, we assumed that other urinary proinflammatory biomarkers may be estimated through measuring plasma proinflammatory biomarkers and would reflect the otherwise undetectable urinary proinflammatory response. Using these methods, we demonstrated multiple examples of reduced ratios of intrarenal urinary anti-inflammatory biomarkers divided by blood proinflammatory biomarkers both pre- and post-operatively in PTOS-AKI. Although not all biomarker ratios were significantly reduced in PTOS-AKI patients, the overall picture is of significant reductions of these ratios in AKI, suggesting that the renal anti-inflammatory response is indeed protective of intrarenal proinflammatory-mediated renal injury arising from a wide range of filtered proinflammatory biomarkers. Additionally, it is known that hypoperfusion and IRI can not only cause direct renal injury but also cause a secondary renal injury through heightened proinflammatory mechanisms (15); this may be why ratios of urinary anti-inflammatory biomarkers/urinary biomarkers of hypoperfusion (H-FABP), and IRI (MK) were also decreased in those who developed PTOS-AKI. This suggests that intrarenal anti-inflammatory responses may provide some protection against collateral proinflammatory renal injury arising from ischemia and IRI.

Our secondary hypothesis was also partially confirmed with reductions observed in PTOS-AKI patients in ratios H2 involving urine anti-inflammatory and blood anti-inflammatory biomarkers. Blood proinflammatory biomarkers drive the blood anti-inflammatory response but are more readily filterable at the glomerulus than their anti-inflammatory biomarker counterparts which are of higher molecular weight (12). Accordingly, blood anti-inflammatory biomarkers which are more consistently retained in plasma than their blood proinflammatory counterparts, are readily available as surrogates for the more difficult to measure blood proinflammatory response. Reductions in ratios of the urine anti-inflammatory response and the blood anti-inflammatory response in PTOS-AKI patients are also consistent with the urinary anti-inflammatory response protecting against renotoxic effects of filtered proinflammatory biomarkers.

Our tertiary hypothesis was also partially confirmed that for patients who develop AKI at orthopaedic fracture surgery, there would be elevated ratios of H3 involving blood anti-inflammatory and blood proinflammatory biomarkers. Greater filtration of blood proinflammatory biomarkers as compared with their blood anti-inflammatory counterparts means that measurement of those unfiltered proinflammatory biomarkers retained in blood fails to quantify the proportion of proinflammatory biomarkers which have already exited the plasma for the tubules. However, if the blood anti-inflammatory response is proportionate to the underlying proinflammatory biomarkers (both filtered and unfiltered), which drive it, then an increase in this ratio would suggest that some proinflammatory biomarkers may have already exited the blood through filtration, thus increasing the ratio. Accordingly, increases in these ratios being associated with PTOS-AKI may be due to proinflammatory biomarkers being filtered from the blood into the kidney where they may exert intrarenal injurious effects.

Lasso regression analyses identified biomarker ratio combinations which could be used to stratify patients at risk of developing PTOS-AKI both pre- and post-operatively. Pre-operatively, a combination of biomarker ratios gave an AUROC of 0.714, which is similar to the AUROC for sTNFR1 previously reported in this patient cohort (13). Post-operatively a combination of biomarker ratios gave an AUROC of 0.837, which is lower than the AUROC of 0.885, previously reported, consisting of H-FABP, sTNFR1 and midkine (13). While the use of biomarker ratios have similar predictive values to biomarker combinations previously published (13), the biomarker ratios also illustrate the potential mechanisms underlying the pathophysiology of PTOS-AKI.

### Limitations of the study and future directions

As reported in our earlier publication, only four of 19 clinical parameters differed between the two groups (13). There were significant differences in age, length of hospital stay, incidence of dementia and hypertension status of the patients who developed PTOS-AKI. These differences may have influenced the development of PTOS-AKI and cytokine response between the study cohorts. Arguably, inclusion of these clinical factors in prospective studies could improve the performance of the biomarker ratio combinations. However, in this study, the emphasis was on proof of concept of biomarker ratio utility. Another limitation for the study was that information on the time from when the patient suffered the initial injury was not available and differences in time from their trauma to surgery may have influenced cytokine response.

As discussed previously (13), patients were assumed to have normal renal function, based on available clinical history prior to their trauma, therefore, patients with undiagnosed renal dysfunction may have been misclassified in the study.

This study has not addressed the practical considerations of introducing the identified biomarker ratios into clinical practice. Both pre- and post-operatively, the highest AUROCs were achieved using a combination of five ratios with some biomarkers measured in urine, plasma and serum. Future research could address the feasibility, and cost-effectiveness of measuring these biomarkers and adoption of the biomarker ratios as a tool to stratify patients at risk of developing PTOS-AKI.

## Conclusions

In summary, in PTOS-AKI patients, the urinary anti-inflammatory response appears to protect against the renal injurious effects of proinflammatory biomarkers, hypoperfusion and IRI. Post-trauma orthopaedic surgery is a more challenging environment than elective surgery to demonstrate biomarkers and combinations which may be predictive of post-operative renal dysfunction. Nevertheless, biomarkers and combinations thereof selected based on their contribution to the pathophysiology of peri-operative renal dysfunction hold promise for informing more effective and earlier management of peri-operative AKI.

## Data Availability

The raw data supporting the conclusions of this article will be made available by the authors, without undue reservation.
